# An outcomes-based module education via flipped classroom enhances undergraduate oral histopathology learning

**DOI:** 10.1186/s12909-023-04753-9

**Published:** 2023-11-09

**Authors:** Yi Zhong, Yuyao Zhang, Wen Sun, Lu Li, Wei Zhang, Yue Jiang, Xiaoqing Lu, Chenjie Cai, Huiling Wang, Laikui Liu, Yan Xu

**Affiliations:** 1 Jiangsu Key Laboratory of Oral Diseases, Nanjing Medical University, Nanjing, China; 2 Department of Basic Science of Stomatology, Affiliated Hospital of Stomatology, Nanjing Medical University, Nanjing, China; 3 Department of Periodontics, Affiliated Hospital of Stomatology, Nanjing Medical University, Nanjing, China

**Keywords:** Outcomes-based education, Oral histopathology, Module, Flipped classroom

## Abstract

**Introduction:**

Oral histopathology is a bridge course connecting oral basic medicine and clinical dentistry. However, the application of outcomes-based education via flipped classroom (FC) in oral histopathology has not been well explored. This study has assessed the efficacy of outcomes-based education via FC in undergraduate oral histopathology module learning in Nanjing Medical University of China.

**Materials and methods:**

A total of 214 third-year students were enrolled and assigned to the FC group of the batch 2022-23 (n = 110) and the traditional classroom (TC) group of the batch 2021-22 (n = 104) to participate the oral histopathology sessions respectively in the study. The FC group were required to preview the online course materials pre-class, followed by in-class quizz, in-class interactive group discussion, and slides microscopic observation. The outcomes-based formative and summative assessments for FC were designed. The TC group attended traditional laboratory class for the same glass slides microscopic observation. In addition, a questionnaire was performed to investigate the satisfaction of learning. Along with this, the performances of FC group in written theory tests and oral histopathology slide tests were compared with TC group.

**Results:**

Students in the FC group gained significantly final higher scores of the course than those in the TC group (score: 83.79 ± 11 vs. 76.73 ± 10.93, *P*<0.0001). Data from the student questionnaires indicated a preference for outcomes-based module education via FC. In the questionnaires, most students considered outcomes-based module education via FC to be beneficial to learning motivation, knowledge comprehension, critical thinking and teamwork. FC group had a higher level of satisfaction with oral histopathology teaching than TC group (satisfaction score: 4.599 ± 0.1027 vs. 4.423 ± 0.01366, *P*<0.01).

**Conclusion:**

An outcomes-based module education via FC has a promising effect on undergraduate oral histopathology education.

**Supplementary Information:**

The online version contains supplementary material available at 10.1186/s12909-023-04753-9.

## Introduction

Oral histopathology is a bridge course connecting oral basic medicine and clinical dentistry and also a compulsory course for undergraduate dental students in China [1]. The main goal of undergraduate oral histopathology teaching is to provide students with an understanding of the nature of diseases. Oral histopathology teaching is a hard, complicated and challenging task [2].

As ‘organising for results’ in various education systems, outcome-based medical education (OBE) was adopted as a framework for the design of undergraduate medical curricula all around the world and used [3]. Emphasis is placed on the intended educational outcomes of thecourses and in turn the educational programme is designed and evaluated considering these educational outcomes [4]. In recent years, as a basic subject for clinical dental diseases learning, the oral histopathology module learning has therefore been developed based on OBE in this study.

Constructivism is an approach to knowledge and learning. It emphasizes the initiative of learners and holds that learning is a process in which learners generate meaning and construct understanding based on their original knowledge and experiences [5]. It has been suggested that constructivism has important guiding value for teaching and learning design [6]. Also, the social constructivist theory has emphasized the importance of interaction between students and teachers to stimulate effective learning [7]. Interaction and collaborative learning online can finally stimulate the development of critical thinking skills, reflection and transformative learning [8]. In the last decades, blended learning has described in-person, classroom-based, synchronous instruction that incorporates elements of online learning, and technology-enhanced pedagogies has been getting more and more attention in academic education [9]. It changes the teaching from teacher-centered to student-centered, increasing interaction between students and teachers for improved learning [10]. The flipped classroom (FC) is an important pedagogical approach that shifts teacher-centered and lecture-based traditional education into student-centered active learning education, and has been gaining popularity in medical education [11]. Blended learning via FC is a typical application of constructivism theory in this study. In the present study, we’ve administered the Super Star Platform and the virtual educational system for undergraduate dental students that will help educators and students as they navigate off-class learning. This study aimed to identify the effectiveness and the students’ satisfactions in the outcomes-based oral histopathology module education via FC in Nanjing Medical University of China.

## Materials and methods

### Participants and setting

This study was conducted at the School of Stomatology of Nanjing Medical University from 2021 to 2023 in China. The oral histopathology curriculum consists of two modules. The module one is oral histology, and oral maxillofacial embryology. The module two is oral pathology, about the etiology, pathogenesis and outcomes of oral diseases. And the oral pathology module content is synchronized with the teaching of corresponding oral diseases (Table 1). 214 dental students of the batch 2021-22 and the batch 2022-23 were invited to participate in oral histopathology course. In their first three years of study, the undergraduate dental students had grasped the basic knowledge of histology, embryology and the subjects of fundamental medicine. All methods were carried out in accordance with relevant guidelines and regulations.

**Table 1:** Syllabus for oral histopathology curriculum

Module	Lesson	Content	Theoretical lessons hours	Lab sessions hours
Module One (36 h, Grade point 2)	Lesson one	Tissues of tooth	5	3
	Lesson two	Periodontal tissues	3	3
	Lesson three	Oral mucosa	2	3
	Lesson four	Salivary glands	2	3
	Lesson five	Oral and maxillofacial development	3	3
	Lesson six	Dental development and dysplasia	3	3
Module Two (50 h, Grade point 2)	Lesson seven	Dental caries	4	3
	Lesson eight	Diseases of pulp and periapical tissues	2	3
	Lesson nine	Periodontal tissue diseases	2	3
	Lesson ten	Oral mucosal diseases	4	3
	Lesson eleven	Oral and maxillofacial cysts	2	3
	Lesson twelve	Odontogenic tumors	4	3
	Lesson thirteen	Salivary gland tumors	4	3
	Lesson fourteen	Other tumors and tumor-like lesions of oral and maxillofacial region	4	3

### Study design

**Fig. 1:**
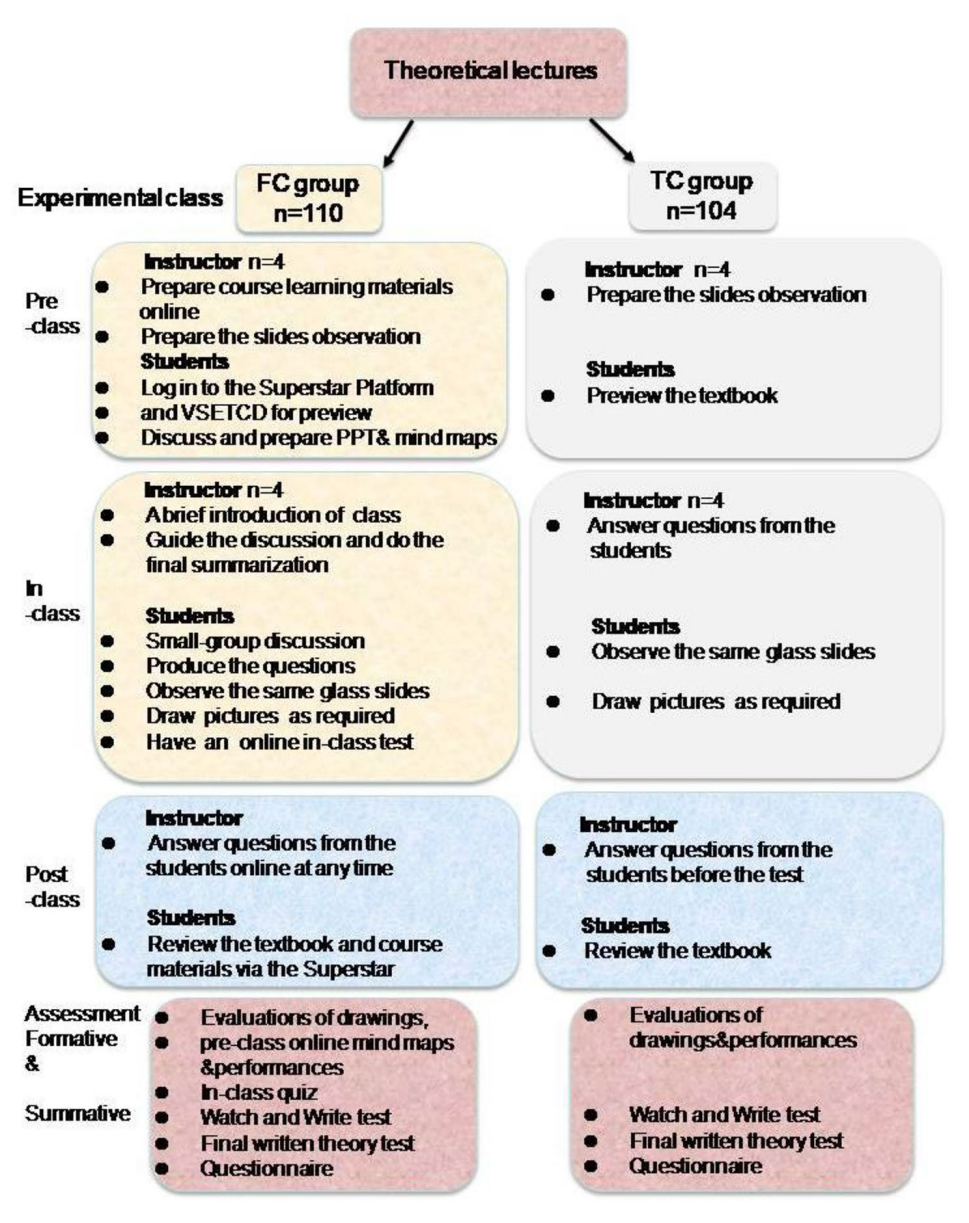
Flow chart of Study methodology

Figure 1 showed the flow chart of the process in this study. Participants were divided into flipped classroom (FC) group of batch 2022-23 (n = 110) and the traditional classroom(TC) group of the batch 2021-22 (n = 104). Due to the intervention within the curriculum, it was not reasonable and justified to identify the control within the same group. Hence students of of the batch 2021-22(n = 104), who were taught using traditional teaching by the same teaching group, were used for comparison. The classes in FC and TL groups were conducted by the same instructor to guarantee the consistency of the teaching content and objectives. Both groups have theoretical lectures by the same teaching group in classroom. In laboratory class phase, participants were assigned to 2 groups: the FC group, wherein participants received FC-based blended study design (n = 110); and the TC group, wherein participants received the traditional experimental study design (n = 104).

**Fig. 2:**
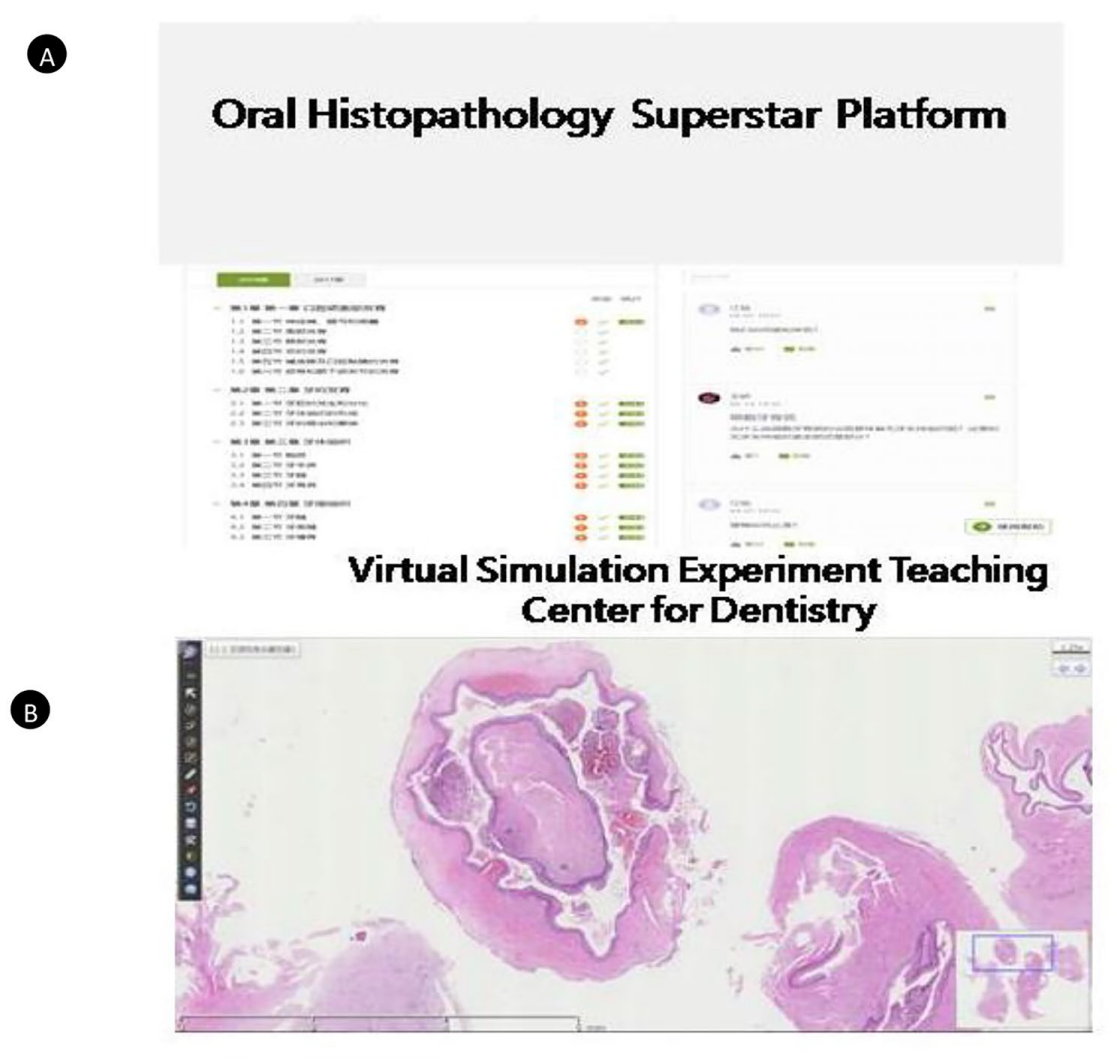
Application of online platforms. (**A**) Application of Superstar platform of NJMU; (**B**) Application of Virtual Simulation Experiment Teaching Center for Dentistry

In the pre-class section of FC laboratory class based on Gagne’s events of instruction [12], the teaching team should formulate content and prepare accurate course learning materials suitable for FC learning modality one week before the class. As shown in Fig. 2A, the Superstar Platform can act as a stage for sharing and discussion. Students logged in to the Superstar Platform to further preview teaching materials for each class. As shown in Fig. 2B, students did virtual learning with virtual microscopic slides supported by Virtual Simulation Experiment Teaching Center for Dentistry (VSETCD) of Nanjing Medical University. Each student was able to visualize the digital slides independently and freely using the VSETCD. At the same time, with teaching content and task-oriented, teachers urge students to carry out discussions in groups independently through online and offline learning modules for pre-study.

The face-to-face in-class laboratory session started with a brief outline of lectures by the instructor, followed by online in-class test serving as one of the formative assessments for evaluating students’ learning efficacy. Then a brief presentation and discussion on students’ own PPTs within pre-assigned groups (n = 6–7) were made. Under the guidance of teachers, students have class discussion and display discussion results in groups face to face. Students were asked to study the learning materials on their own time and prepare their PPTs to explain the learning points. Students in groups then collaborated to take turns interpreting and discussing the slides and cases proposed by teachers. Also teachers can summarize the main knowledge points and answer all questions as feedbacks for the FC discussion. Students are required to use optical microscope to observe characteristics of the stained slides and draw pictures by themselves. Teachers should mark the attendances and performances of the students. After oral histopathology laboratory classes, teachers assigned homework on Superstar Platform and the students complete it in time. Based on students’ learning interests, the latest and related dental research results were also supplemented on Superstar Platform by teachers to broaden knowledge. When students left a message for discussion on Superstar Platform, the teacher should give timely help and feedbacks.

The rest 104 participants in TC group attended the laboratory class and used microscope to observe the same glass slides for morphological learning. Also students were required to observe characteristics and draw pictures by themselves. Finally, teachers arranged written theory tests and oral histopathology slide tests (Watch and Write test) as laboratory tests for two groups.

### Course assessments

The corresponding participants’ assessments for FC based on OBE have also been designed. The assessments for FC consisted of the formative assessment for daily performance (25%), in-class quiz score (5%), and summative assessment for laboratory slides test (Watch and Write test) score (10%) and final written theory test score (60%). The formative assessment for daily performance included the evaluations of students’ drawings, mind maps and performances in laboratory class. In the laboratory course, students should observe sections with a microscope and draw the microscopic manifestations of typical section to deepen their understandings of typical structures through drawing, making up 10 per cent of the total score. The scores for pre-class online mind map assignment made up 6 per cent of the total score. In-class oral presentation made up 9 per cent of the total score. The laboratory slides test evaluated students’ grasping of structures, making up 10 per cent of the total score. In-class quiz were also arranged as stage evaluation, making up 5 per cent of the total score. The in-class quiz included 10 multiple choice questions and should be answered in 5 min. The final written theory test included multiple choices and short answer questions, making up 60 per cent of the total score, mainly covering theoretical knowledge of oral histopathology. Tests were designed by a non-investigator from a third party based on the teaching syllabus. The participants’ assessments for TC were students’ drawings assessment (10%), laboratory test (10%), and final written theory test (80%). The question types of the exams of TC were the same as FC. The identity information of any participant was covered to avoid bias, and scores were double-checked by two examiners.

### Questionnaire survey

A questionnaire after class was conducted with the participants at the end of the semester to demonstrate the feedbacks towards learning experience. The questionnaire points per subject were obtained. Responses were sorted and analyzed by two independent researchers (1 = strongly disagree, 2 = somewhat disagree, 3 = neither agree nor disagree, 4 = somewhat agree, and 5 = strongly agree). The participants provided informed written consent, and the study followed the Declaration of Helsinki and the guidelines of the Ethics Review Committee of Affiliated Stomatological Hospital of Nanjing Medical University with regard to medical protocols and ethics.

### Statistical analysis

Statistical analysis was performed by the statistical software program SPSS 22.0 for Windows software (SPSS, Inc., Chicago, IL, USA). The scores of 5-point Likert scale in the survey were compared between the 2 groups by nonparametric Mann–Whitney Test. The scores were analyzed by independent samples t test. The χ^2^ test was used to analyze the sex and nationality match. The statistical data were presented as mean ± standard error of the mean (SEM). *P*<05 was defined statistically significant.

## Results

### Baseline data

A total of undergraduate dental students, aged from 21 to 25 years old, were recruitedin the study, and none of the students were missing at different stages of the study. The sample was composed of dental students in FC group (n = 110) and TL group (n = 104). There were no significant differences in terms of nationality and sex between two groups (Table 2).

**Table 2:** Demographic information of participants in the study

Number of participants (percentage)		
	TC(n = 104)	FC(n = 110)
Gender*		
Male	35(33.65%)	41(37.27%)
Female	69(66.35%)	69(62.73%)
Nationality^#^		
Han	102(98.08%)	106(96.36%)
Ethnic minority	2(1.92%)	4(3.64%)

FC: flipped classroom; TC: traditional classroom

*: ns *P* = 0.6683(value of gender); #: ns *P* = 0.6838(value of ethnic)

### Students’ performances in the assessments

**Fig. 3:**
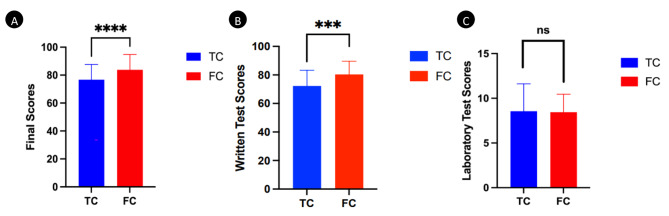
Outcomes-based assessments after the course **A**: The comparison of final scores between the FC group and TC group; **B**: The comparison of written test scores between the FC group and TC group; **C**: The comparison of laboratory test scores between the FC group and TC group. ***: *P*<0.001; ****: *P*<0.0001

The final scores of the FC group were significantly higher than those of the TC group (score: 83.79 ± 11 vs. 76.73 ± 10.93, *P*<0.0001, Fig. 3A). The final written test scores of the FC group were significantly higher than those of the TC group (score: 80.37 ± 9.28 vs. 72.32 ± 10.93, *P*<0.001, Fig. 3B). Furthermore, the students’ laboratory test scores of FC group were significantly higher than those of TC group (score: 9.07 ± 7.04 vs. 8.83 ± 4.96, *P*>0.05, Fig. 3c).

The percentage of high total test score (test score>85) of the FC group was also significantly higher than that of the TC group (n = 126, 38% vs.n = 19, 18%, *P*<0.001). The percentage of low total test score (test score<70) showed no difference between the FC group and the TC group (n = 18, 16% vs.n = 24, 23%, *P*>0.05). There were no differences of the drawing scores between the FC group and the TC group (score: 7.35 ± 1.73 vs. 6.58 ± 1.27, *P*>0.05).

The mean final scores of the FC group were significantly higher than those of the TC group in module one(score: 84.25 ± 8.57 vs. 74.40 ± 12.81, *P*<0.001). The mean final scores showed no difference between the FC group and the TC group inmoduletwo (score: 78.64 ± 10.0 vs. 79.01 ± 8.14, *P*>0.05).

### Students’ attitudes in the questionnaires

**Fig. 4:**
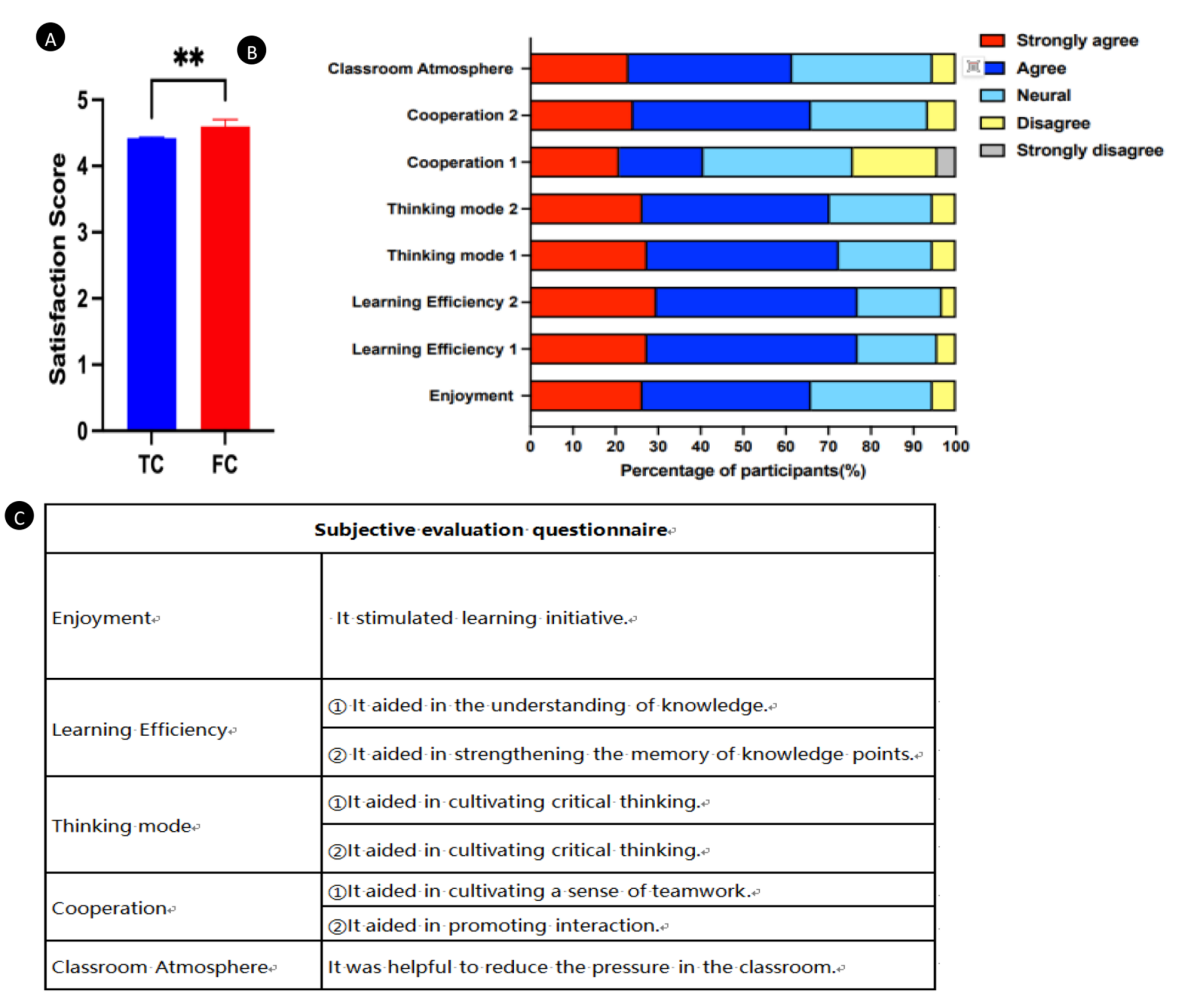
Students’ perceptions and comments of the course **A**: The comparison of satisfaction scores in teaching between the FC group and TC group; **B**: The satisfaction rate towards FC teaching modality; **C**: The questionnaire concerning FC. *P*＜0.01

All the participants were asked to rate their perceptions of learning at the end of the course. The surveys of oral histopathology learning were tailored for the students in FC and TC group respectively. All data were collected and analysed. The positive responses led to a higher rate of satisfaction with FC group than TC group (satisfactionscore: 4.599 ± 0.1027 vs. 4.423 ± 0.01366, *P*<0.01, Fig. 4A). Moreover, students who participated in this study finished the online questionnaires on their self-perceived competence and opinions towards FC teaching modality. In the questionnaires, most students considered outcomes-based module education via FC to be beneficial to learning motivation, knowledge comprehension, critical thinkingand teamwork. As shown in Fig. 4B-C, nearly 65.94% of the students strongly agreed that they could enjoy learning via FC. 76.92% out of students agreed that it makes easier for them to understand the lessons compared with the conventional method. This study revealed that 54.81% out of students agreed that FC significantly improved students’ teamwork and interaction. 61.54% of the students agreed that classroom learning pressure is reduced via FC.

## Discussion

Previous studies have shown that student-centred teaching can cultivate active thinking, problem solving and provide feedback on the learning process [13]. Thus, various student-centred teaching methods have been conducted in medical teaching and learning [14, 15]. Presently, the traditional teaching model in medicine curricula is still the lecture-based classroom and students’ in-class listening model. As medical education reform is deepen, the hybrid teaching models online and offline are gradually gaining attention. In recent years, an outcomes-based undergraduate oral histopathology module learning design via FC has innovatively been conceived in this study, following the cognitive constructivism educational theory [6]. The FC process was administered according to the guidelines described in previous literature [16].

Blended learning breaks the constraints of time and space, creates flexible, rich and coherent learning experiences, and brings about fundamental changes in teaching methods and structures in a new era [17]. It has been proposed to adopt the combination of online and offline teaching methods to practice in the teaching of oral histopathology modules in this study. Blended learning contributes more to knowledge acquisition than face-to-face and online education respectively [18]. This whole process can stimulate students’ interest in learning and cultivate students’ enthusiasm for learning [19]. It turns out the similar results confirmed by the FC-based blended learning conducted in this study. Moreover, the ‘FC’ concept, which indicates face-to-face teacher instructions are replaced with individual or group homework activities, is often used in blended learning [20]. We can see an improvement of students’ teamwork ability was found with the application of FC in this study. Compared to TL, where there is only teacher-student interaction, the in-class study in FC with both teacher–student interaction and student-student interaction may contribute to promoting the teamwork capability, which is of significance for medical students because hospitals are workplaces based on teamwork [21]. And what’s more, the FC-based blended learning implemented has also improved student’s learning innovation and critical thinking in this study. By previewing the slices and related theories of the laboratory lessons in advance, students can increase active learning and knowledge internalization. It is true that students have been allowed to construct and gain knowledge by active learning and exchanging views.

FC is a good pedagogical approach for student-centered active learning education [20]. It has been confirmed that more activelearning in FC can result in a better learning result [22]. Also, FC in health professionals showed improved student learning in one recent meta analysis [23]. In this study, the outcomes-based module learning via FC has enhanced students’ better understandings of oral disease and their initiatives of learning. Through the learning, undergraduate dental students’ learning efficiency of oral histopathology has increased. It has shown a statistically significant increase in mean final scores of the FC group. For years, oral histopathology has developed from a macroscopy and autopsy based discipline to a finessed histological and molecular field with great advances [1, 2]. The FC-based blended teaching via Superstar Platform enables students to preview and review the knowledge of oral histopathology besides face-to-face laboratory teaching, which is conducive to improving learning efffciency.

During the outcomes-based module learning via FC in this study, formative assessments have been strengthened. Formative assessment and summative assessment can both provide professional assessments and regulation to improve the teaching and learning efficiency. On one hand, formative assessment can inspire them to learn and reinforce students’ activations during the study period. On the other hand, summative assessment can make the overall judgment of the students’ performances [24, 25]. The most common strategy is to provide students with regular assessments of their knowledge by either summative or formative assessments [26]. In this study, the participants’ assessments for FC consist of the formative assessment and summative assessment. A summative assessment was conducted among allenrolled students by the laboratory and written theories tests, which were organized after the teaching in this study. Consequently, the final written test as summative assessment outcomes of the FC group were significantly better than the TC group in this study. The reason for no significant difference in laboratory test maybe that the Watch and Write test as summative assessment is relatively simple. And the number for Watch and Write test is relatively small, not covering all the knowledge aspects. The formative assessment for daily performances included the evaluations of students’ drawings, online mind maps, performances in laboratory class and quizzes in class. Numerous studies have shown that regular assessments are beneficial to students’ learning [27]. Formative assessment can objectively evaluate students’ learning attitude and learning efficiency. It has shown that the mean final scores of the FC group were significantly higher than those of the TC group.

In this study, the majority of students were satisfied with FC-based blended learning based on the questionnaire answers. More students from FC group felt satisfied with the adopted FC-based teaching model than those from TL group. Also, blended learning has the potential to improve clinical competencies [28]. It has been reported that multi-mode teaching methods including FC play their vital roles in the life-long career development of medical students [29]. Coincidentally, this study showed that the cultivation of clinical thinking of the FC group was enhanced, consistent with the literature. Thus, long-term assessments are promising in the future clinical dentistry. Additionally, teachers can summarize the main knowledge points and answer all questions for the in-class FC discussion. During the course, students are more interactive and engaged in the FC group, and teachers can see teaching blind spots and other areas for improvement and learning knowledge points which should be emphasized. Indeed, an improvement of interaction with the students was described in this study. Thus teachers should constantly improve teaching methods and adjust teaching content so as to increase academic level and teaching efficiency. It has been found that the mean final scores of the FC group were significantly higher than those of the TC group in module one, but showed no difference from the TC group in module two. Reflection on the feedbacks from students suggest that more clinical elements and corresponding case-based learning should be added for module two of oral pathology.

Thus in this study, knowledge learning assessment is still the main part of the total assessment. FC can contribute better to knowledge teaching and learning efficiency, in line with the literature [30, 31]. In conclusion, this outcomes-based module education via FC has enhanced undergraduate oral histopathology learning and will be a promising education model in the future. We suggest that outcomes-based module education via FC can provide students with education that goes beyond existing education.

### Study limitations

However, this study involved a small number of dental students from a single institution.There have been a few students staying neutral about FC. The learning of oral histopathology should not be just assessed by short term performances. Future dental clinical performances should be followed. More work should explore the systematic assessment and timely feedbacks of learning. Outcomes-based module learning via FC and constant feedbacks should be established and integrated into undergraduate oral histopathology education.

## Conclusion

To sum up, this study suggest that implementation of the outcomes-based module education via FC in oral histopathology have a positive impact on students’ performance and appreciated by students. Therefore, the findings highlight the application of outcomes-based module education via FC will represent a promising tool for traditional dental education in the future.

### Electronic supplementary material

Below is the link to the electronic supplementary material.

Supplementary Material 1

Supplementary Material 2

Supplementary Material 3

Supplementary Material 4

## Data Availability

The authors declare that all the data and materials supporting the findings of this study are available within the article and are available from the corresponding author upon request.

## List of Abbreviations

FC: Flipped classroom
TC: Traditional classroom
VSETCD: Virtual Simulation Experiment Teaching Center for Dentistry
