# Supplementary material for: An outcomes-based module education via flipped classroom enhances undergraduate oral histopathology learning
Source: BMC Med Educ. 2023 Nov 9;23:848. doi: 10.1186/s12909-023-04753-9 (PMC10637004; doi:10.1186/s12909-023-04753-9)
Supplement: Supplementary file 1 — Supplementary Material 1 [file 12909_2023_4753_MOESM1_ESM.docx]

**Appendix 1: Gagne Lesson Plan Template**

Session title: Oral histopathology Laboratory teaching

Student / trainee level: Undergraduate dental students

| **Level** | | **Activity** |
| --- | --- | --- |
| 1 | Gaining attention | Teachers finish roll call and begin the class. |
| 2 | Informing learner of objectives | In the laboratory class, the teacher re-emphasizes the learning objectives of this chapter.  For example, the learning objectives for oral and maxillofacial cysts are as follows:  1）Describe pathological showings of odontogenic keratocysts, dentigerous cysts and mucinous cysts.  2）Discuss the occurrence and diagnosis process of odontogenic cysts. |
| 3 | Stimulate recall of prior learning | 1.Before the watching study of pathological sections of cysts in the oral and maxillofacial region, teachers should ask questions about the related content of the lesson, and the students freely raise their hands to answer. For example, what are the characteristics of oral and maxillofacial development？Moreover, why are cysts prone to occur in the oral and maxillofacial region?  2.In face to face class, students display summary of learning outcomes for the questions on Superstar Platform in the form of PPTs, videos in groups. |
| 4 | Presenting stimulus | 1.During group discussions, all students can ask each other any related question concerning the class, evaluate the performance of each other, and seek help from the teacher if they have difficulty in solving problems.  2.Next teachers give comments and summary, stimulating students' interest in the teaching content. |
| 5 | Providing learning guidance | In the demonstration teaching, teachers make PPTs presentation and operate the procedures of watching the pathological sections, explain the key points.  For example, students firstly can find the cyst cavity under low magnification, and focus on the epithelial lining and fibrous walls surrounding the cyst cavity under high magnification. |
| 6 | Eliciting performance | During the experiment course, students observe pathological sections under a microscope by themselves. If students have any questions, they can raise their hands and ask the teacher for help. |
| 7 | Providing feedback | Teachers should encourage students to actively raise their own doubts and point out operational errors and knowledge blind spots in time. |
| 8 | Assessing performance | 1.After watching the pathology sections, students presented typical sections and invited classmates to answer related questions. Students can comment on each other.  2.Also, teachers emphasize key knowledge points and give comments concerning the students’ performances such as classroom attendance, participation in practical activities, etc.  3.Furthermore, the teacher assign in-class quiz on Superstar platform and the students complete homework in time. |
| 9 | Enhancing retention and transfer | 1.The Watch and Write test is specifically designed for oral histopathology laboratory class. The Watch and Write test will be assessed by teachers.  2.The teaching effectiveness was also evaluated by questionnaire survey concerning the teaching at the end of the semester.  3.When students leave a message about the teaching and learning on Superstar Platform, the teacher should give timely feedback. Students should be informed of the results about the learning process in time.  4.Teachers will analyze the causes of the common mistakes for students and give the feedbacks , stressing students' motivation to learn. |
