# Supplementary material for: An outcomes-based module education via flipped classroom enhances undergraduate oral histopathology learning
Source: BMC Med Educ. 2023 Nov 9;23:848. doi: 10.1186/s12909-023-04753-9 (PMC10637004; doi:10.1186/s12909-023-04753-9)
Supplement: Supplementary file 2 — Supplementary Material 2 [file 12909_2023_4753_MOESM2_ESM.docx]

**Questionnaire Towards Flipped Classroom in Oral Histopathology Teaching**

| **Questions Answers Grades** | |
| --- | --- |
| Do you agree that flipped classroom has stimulated learning initiative? | Strongly agree |
|  | Agree |
|  | Neural |
|  | Disagree |
|  | Strongly disagree |
| Do you agree that flipped classroom has aided in the understanding of knowledge? | Strongly agree |
|  | Agree |
|  | Neural |
|  | Disagree |
|  | Strongly disagree |
| Do you agree that flipped classroom has aided in strengthening the memory of knowledge points? | Strongly agree |
|  | Agree |
|  | Neural |
|  | Disagree |
|  | Strongly disagree |
| Do you agree that flipped classroom has aided in cultivate critical thinking? | Strongly agree |
|  | Agree |
|  | Neural |
|  | Disagree |
|  | Strongly disagree |
| Do you agree that flipped classroom has aided in cultivate clinical thinking? | Strongly agree |
|  | Agree |
|  | Neural |
|  | Disagree |
|  | Strongly disagree |
| Do you agree that flipped classroom has aided in cultivate a sense of teamwork? | Strongly agree |
|  | Agree |
|  | Neural |
|  | Disagree |
|  | Strongly disagree |
| Do you agree that flipped classroom has aided in promoting interaction? | Strongly agree |
|  | Agree |
|  | Neural |
|  | Disagree |
|  | Strongly disagree |
| Do you agree that flipped classroom been helpful to reduce the pressure in the classroom? | Strongly agree |
|  | Agree |
|  | Neural |
|  | Disagree |
|  | Strongly disagree |

What else do you feel could be improved? Considering the above, what are the other suggestions for future course study?

Name Gender Date
