# Supplementary material for: An outcomes-based module education via flipped classroom enhances undergraduate oral histopathology learning
Source: BMC Med Educ. 2023 Nov 9;23:848. doi: 10.1186/s12909-023-04753-9 (PMC10637004; doi:10.1186/s12909-023-04753-9)
Supplement: Supplementary file 3 — Supplementary Material 3 [file 12909_2023_4753_MOESM3_ESM.docx]

**Questionnaire Towards Oral Histopathology Module Learning**

| **Questions Answers Grades** | |
| --- | --- |
| Do you agree that oral histopathology module learning  has improved learning efficiency? | Strongly agree |
|  | Agree |
|  | Neural |
|  | Disagree |
|  | Strongly disagree |
| Do you agree that oral histopathology module learning  has been beneficial to the dental clinical course study? | Strongly agree |
|  | Agree |
|  | Neural |
|  | Disagree |
|  | Strongly disagree |
| Do you agree that oral histopathology module learning  has been helpful for the comprehension and memorization of oral diseases? | Strongly agree |
|  | Agree |
|  | Neural |
|  | Disagree |
|  | Strongly disagree |
| Do you agree that oral histopathology module learning  has contributed to enlightening clinical critical thinking? | Strongly agree |
|  | Agree |
|  | Neural |
|  | Disagree |
|  | Strongly disagree |
| Do you agree that oral histopathology module learning  has improved learning efficiency? | Strongly agree |
|  | Agree |
|  | Neural |
|  | Disagree |
|  | Strongly disagree |
| Do you agree that oral histopathology module learning  has guided students’ thinking? | Strongly agree |
|  | Agree |
|  | Neural |
|  | Disagree |
|  | Strongly disagree |
| Do you feel satisfied with oral histopathology learning? | Very much |
|  | General |
|  | Neural |
|  | A little |
|  | Bad |

What else do you feel could be improved? Considering the above, what are the other suggestions for future course study?

Name Gender Date
