# Supplementary material for: An outcomes-based module education via flipped classroom enhances undergraduate oral histopathology learning
Source: BMC Med Educ. 2023 Nov 9;23:848. doi: 10.1186/s12909-023-04753-9 (PMC10637004; doi:10.1186/s12909-023-04753-9)
Supplement: Supplementary file 4 — Supplementary Material 4 [file 12909_2023_4753_MOESM4_ESM.docx]

**Assessment of the Questionnaire Towards Flipped Classroom in Oral Histopathology Teaching**

| **Question Categorization Question Detail Answer Grade** | | | **Percent** |
| --- | --- | --- | --- |
| Enjoyment | It stimulated learning initiative. | Strongly agree | 26.37% |
|  |  | Agree | 39.56% |
|  |  | Neural | 28.57% |
|  |  | Disagree | 5.49% |
|  |  | Strongly disagree | 0.00% |
| Learning Efficiency | ① It aided in the understanding of knowledge. | Strongly agree | 27.47% |
|  |  | Agree | 49.45% |
|  |  | Neural | 18.68% |
|  |  | Disagree | 4.40% |
|  |  | Strongly disagree | 0.00% |
|  | ② It aided in strengthening the memory of knowledge points. | Strongly agree | 29.67% |
|  |  | Agree | 47.25% |
|  |  | Neural | 19.78% |
|  |  | Disagree | 3.30% |
|  |  | Strongly disagree | 0.00% |
| Thinking mode | ①It aided in cultivate critical thinking. | Strongly agree | 27.47% |
|  |  | Agree | 45.05% |
|  |  | Neural | 21.98% |
|  |  | Disagree | 5.49% |
|  |  | Strongly disagree | 0.00% |
|  | ②It aided in cultivate clinical thinking. | Strongly agree | 26.37% |
|  |  | Agree | 43.96% |
|  |  | Neural | 24.18 |
|  |  | Disagree | 5.49% |
|  |  | Strongly disagree | 0.00% |
| Cooperation | ①It aided in cultivate a sense of teamwork. | Strongly agree | 20.88% |
|  |  | Agree | 19.78% |
|  |  | Neural | 35.16% |
|  |  | Disagree | 19.78% |
|  |  | Strongly disagree | 4.40% |
|  | ②It aided in promoting interaction. | Strongly agree | 24.18% |
|  |  | Agree | 41.76% |
|  |  | Neural | 27.47% |
|  |  | Disagree | 6.59% |
|  |  | Strongly disagree | 0.00% |
| Classroom Atmosphere | It was helpful to reduce the pressure in the classroom. | Strongly agree | 23.08% |
|  |  | Agree | 38.46% |
|  |  | Neural | 32.97% |
|  |  | Disagree | 5.49% |
|  |  | Strongly disagree | 0.00% |
